# Does inflammation markers or treatment type moderate exercise intensity effects on changes in muscle strength in cancer survivors participating in a 6-month combined resistance- and endurance exercise program? Results from the Phys-Can trial

**DOI:** 10.1186/s13102-023-00617-3

**Published:** 2023-01-19

**Authors:** Anna Henriksson, Emelie Strandberg, Andreas Stenling, Anne-Sophie Mazzoni, Katarina Sjövall, Sussanne Börjeson, Truls Raastad, Ingrid Demmelmaier, Sveinung Berntsen, Karin Nordin

**Affiliations:** 1 Department of Public Health and Caring Sciences, Uppsala University, Uppsala, Sweden; 2 Department of Sport Science and Physical Education, University of Agder, Kristiansand, Norway; 3 Department of Psychology, Umeå University, Umeå, Sweden; 4 Faculty of Health Sciences, Kristianstad University, Kristianstad, Sweden; 5 Department of Oncology and Department of Health, Medicine and Caring Sciences, Linköping University, Linköping, Sweden; 6 Department of Physical Performance, Norwegian School of Sport Science, Oslo, Norway

**Keywords:** Androgen deprivation therapy, Cancer, Chemotherapy, Exercise intensity, Moderators, Inflammation, Resistance training

## Abstract

**Background:**

Resistance exercise has a beneficial impact on physical function for patients receiving oncological treatment. However, there is an inter-individual variation in the response to exercise and the tolerability to high-intensity exercise. Identifying potential moderating factors, such as inflammation and treatment type, for changes in muscle strength is important to improve the effectiveness of exercise programs. Therefore, we aimed to investigate if inflammation and type of oncological treatment moderate the effects of exercise intensity (high vs. low-moderate) on muscular strength changes in patients with breast (BRCA) or prostate cancer (PRCA).

**Methods:**

Participants with BRCA (*n* = 286) and PRCA (*n* = 65) from the Physical training and Cancer study (Phys-Can) were included in the present study. Participants performed a combined resistance- and endurance exercise program during six months, at either high or low-moderate intensity. Separate regression models were estimated for each cancer type, with and without interaction terms. Moderators included in the models were treatment type (i.e., neo/adjuvant chemotherapy—yes/no for BRCA, adjuvant androgen deprivation therapy (ADT)—yes/no for PRCA)), and inflammation (interleukin 6 (IL6) and tumor necrosis factor-alpha (TNFα)) at follow-up.

**Results:**

For BRCA, neither IL6 (*b* = 2.469, 95% CI [− 7.614, 12.552]) nor TNFα (*b* = 0.036, 95% CI [− 6.345, 6.418]) levels moderated the effect of exercise intensity on muscle strength change. The same was observed for chemotherapy treatment (*b* = 4.893, 95% CI [− 2.938, 12.724]). Similarly, for PRCA, the effect of exercise intensity on muscle strength change was not moderated by IL6 (*b* = − 1.423, 95% CI [− 17.894, 15.048]) and TNFα (*b* = − 1.905, 95% CI [− 8.542, 4.732]) levels, nor by ADT (*b* = − 0.180, 95% CI [− 11.201, 10.841]).

**Conclusions:**

The effect of exercise intensity on muscle strength is not moderated by TNFα, IL6, neo/adjuvant chemotherapy, or ADT, and therefore cannot explain any intra-variation of training response regarding exercise intensity (e.g., strength gain) for BRCA or PRCA in this setting.

*Trial registration*: ClinicalTrials.gov NCT02473003.

**Supplementary Information:**

The online version contains supplementary material available at 10.1186/s13102-023-00617-3.

## Background

The advances in cancer diagnostics and treatment have led to improved survival rates [1], which, in turn, has led to an increased number of people living with side effects from cancer and its treatments. Side-effects such as cancer-related fatigue and poorer health-related quality of life are often reported [2]. Side-effects of cancer treatment may also resemble the natural processes of aging, such as reduced muscle strength and mass, although this reduction is augmented in patients with cancer [3] and appears in both women with breast cancer and in men with prostate cancer [4, 5]. In the general population, poor lower body muscle function is related to reduced function and lower quality of life [6]. In addition, loss of muscle function is associated with mobility disorders, increased risk of falls and fractures, impaired ability to perform activities of daily living, disabilities, loss of independence, and increased mortality [4, 5].

Women who receive chemotherapy for breast cancer may be at a greater risk of declines in muscle strength as chemotherapy may have a direct negative effect on skeletal muscle. For instance, anthracyclines impair the force-generating capacity and mitochondrial function of skeletal muscles in rats [7–10], and these results have been confirmed in two small-scale studies on patients with breast cancer undergoing chemotherapy [11, 12]. Androgen deprivation therapy (ADT) is provided to men with locally advanced prostate cancer to reduce recurrence after curative radiotherapy [13, 14]. However, due to marked reductions in testosterone levels, patients receiving ADT lose approximately 2–4% lean mass during the first year of therapy [15], and this decrease in lean mass is often accompanied by a decrease in strength and physical function [16]. In addition, cancer and its treatments have a negative impact on the systemic milieu, where an increase in inflammation is often observed [17, 18]. Higher levels of systemic inflammation are associated with lower levels of muscle mass and strength [19]. Specifically, pro-inflammatory cytokines, such as tumor necrosis factor-alpha (TNFα) and interleukin (IL)-6, have been demonstrated to increase muscle atrophy and contractile dysfunction of skeletal muscle [20, 21]. The specific inflammatory drivers of muscle dysfunction remain poorly defined, but a suppression of the muscle protein synthesis pathway by inflammation has been suggested [22].

**Fig. 1:**
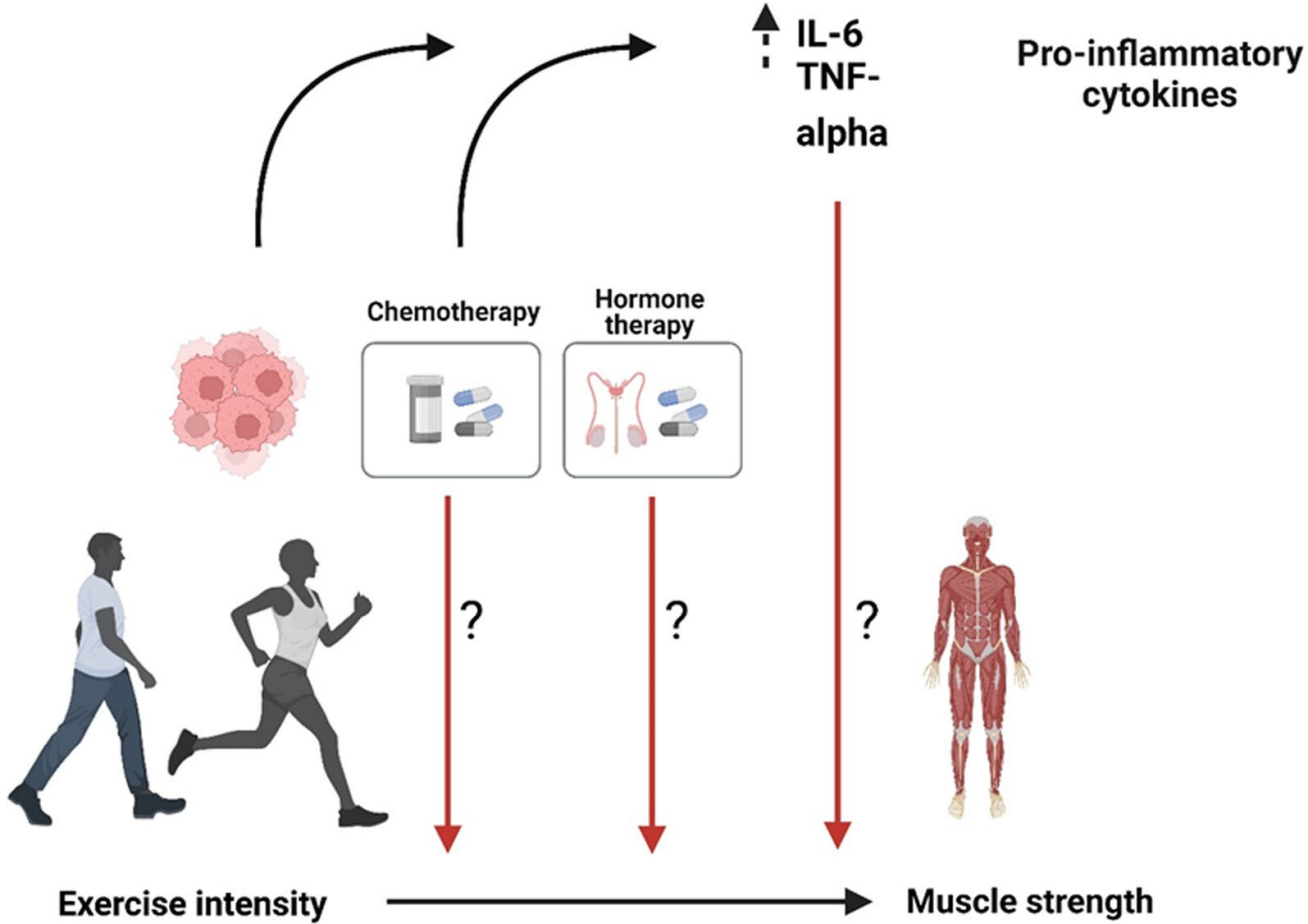
Possible moderators of exercise intensity on strength changes in patients with breast or prostate cancer. IL-6: interleukin 6, TNF- alpha (TNFα) tumor necrosis factor-alpha. Figure was created with BioRender.com

Participating in structured exercise programs during cancer treatment has beneficial effects on cancer-related fatigue [23], muscle fiber size [24], and inflammation [25]. Specifically, supervised resistance training can counteract the loss of muscle mass and improve upper and lower body strength in cancer survivors [26, 27]; however, there are inter-individual variations in strength gains among patients with cancer [26, 27]. Moreover, a recent meta-analysis found that untrained individuals demonstrate higher levels of muscle hypertrophy than individuals with longer experience in resistance training, thus supporting the principle of diminishing returns regarding hypertrophy [28]. Interestingly, individuals with previous training experience demonstrate superior strength gains when increasing exercise sessions [28], although this may be due to a higher exercise volume in response to an increased exercise frequency [29]. For healthy populations, exercising at a higher intensity gives superior improvements in muscle strength in comparison to exercising at a lower intensity, even when the training load is controlled for [29]. Nonetheless, whether the same response to exercise intensity can be achieved for patients undergoing neo- or adjuvant cancer treatment has been less studied. Previous results from one randomized trial (The Phys-Can study), conducted by our research group, showed higher gains in leg strength for participants exercising at high intensity than low-moderate intensity (adjusted mean difference 3.98 [95% CI 0.58–7.38] kg) for cancer patients [30]. In addition, other factors may also influence the training response; consequently, identifying such factors is important to further understand how to improve the effectiveness of exercise programs for patients with cancer. The effects of the systemic milieu (i.e., inflammation) may directly interfere with the molecular response of the muscle to the mechanical stimuli, negatively affecting the training response. In addition, the type of oncological treatment may also influence the training response; however, the moderating effects of inflammation and treatment on changes in muscle strength have not been explored. Therefore, the aim of the present study was to investigate if markers of inflammation and type of neo- or adjuvant cancer treatment moderate the effects of exercise intensity on strength changes in patients with breast or prostate cancer. We hypothesized that increased levels of inflammation and receiving chemotherapy or ADT moderate the effects of exercise intensity on strength for patients receiving neo-or adjuvant treatment (Fig. 1).

## Methods

This study is based on data from participants with breast or prostate cancer included in the Physical training and Cancer (Phys-Can) multicenter randomized controlled trial, which has been previously described in detail elsewhere [30]. Briefly, participants were randomized to either high intensity (HI) or low-to-moderate intensity (LMI) exercise, with or without additional behavior change support (ClinicalTrials.gov NCT02473003). Previous analysis found no effects of behavior change support on exercise adherence [31], but there was a difference in exercise intensity regarding muscle strength change. We therefore conducted the analyses by merging the groups receiving or not receiving additional behavioral change support; thus, the analysis in the present study is based on two groups, HI or LMI exercise. The study was approved by the Swedish Ethical Review Authority (Dnr 2014/249).

### Participants

Included participants were: 1) women with localized or regional stage breast cancer who planned to undergo curative oncological treatment with one or a combination of the following treatments- neo- or adjuvant chemotherapy, adjuvant radiotherapy, or adjuvant endocrine treatment; and 2) men with localized or regional stage prostate cancer who planned to undergo curative radiotherapy with or without neo/adjuvant endocrine treatment (i.e., ADT). Exclusion criteria were conditions that prevented exercise at a high intensity (e.g., severe heart failure, late stages of chronic obstructive pulmonary disease and orthopedic conditions) and/or prevented informed consent (e.g., severe psychiatric disease, cognitive impairments). All participants were assessed by a physician regarding eligibility prior to inclusion [30]. Eligible patients were included and provided written consent before the baseline data collection.

### Exercise intervention

The intervention, consisting of a combined resistance and endurance exercise program with or without addition of behavior change techniques, has been described in detail elsewhere [30]. The supervised resistance training was performed twice per week during six months. The resistance training comprised of six exercises: seated leg-press, seated chest-press, seated leg-curl, seated row, seated leg-extension (pin-loaded) and seated overhead-press (using dumbbells). The training load was based on 6 and 10 repetition maximum (RM) testing. Participants in the HI group performed 3 sets of either 6 RM (once a week) or 10 RM (once a week) with the third (i.e., the last) set being performed until failure. Participants in the LMI group performed 3 sets of either 12 repetitions (once a week) or 20 repetitions (once a week) with a corresponding training load of 50% of 6 and 10 RM, respectively. Importantly, the participants in the LMI group did not perform their last set until failure. Participants also performed home-based endurance training. After an initial warm-up of 10 min at a moderate intensity, the HI group performed 5 × 2-min intervals of running or cycling at 80–90% of individual heart rate reserve (HRR) twice a week, with a progression from 5 to 10 intervals per session over the 6-month intervention. Between each interval, there was a 2-min active rest period. The LMI group performed continuous-based exercise, mainly walking or biking, in bouts of at least 10 min at an exercise intensity of 40–50% of the HRR with the goal being reaching 150 min/week. To reduce risk of negative interference between endurance and resistance training [32], the participants was instructed to perform the endurance training and resistance training on separate days.

### Measures

#### Muscle strength

The participants’ muscle strength was assessed using 1 RM test at baseline and after the exercise intervention, at 6 months. The test at baseline was performed after a 6-week familiarization period and was assessed in seated chest-press and seated single-leg press (left and right leg tested separately). Briefly, following a warm-up period, the load was set at 90–95% of the estimated 1 RM and increased with approximately 2.5–5% after each successful lift until failure. A 3-min resting period was allowed between consecutive attempts, and 1 RM was obtained within five attempts [30]. For the analysis, results from the single-leg press (right and left leg) were summed together and expressed as total leg strength.

#### Inflammation

Blood samples were obtained at baseline and after 6 months. Participants were asked to avoid smoking and alcohol and not to engage in any strenuous physical activity 24 h before the blood sampling. Venous blood was collected in EDTA tubes, centrifuged at 2400 g for seven minutes, followed by plasma isolation. Plasma was stored at each study site at − 80 °C until transported to the Centre for Physical Activity Research at the University of Copenhagen in Denmark for analysis. The analysis of Interleukin 6 (IL-6) and tumor necrosis factor-alpha (TNFα) was measured using the pro-inflammatory panel 1 kit (V-Plex technology, Meso Scale Discovery, Rockville, MD, U.S.A.) according to the manufacturer’s guidelines as previously described [33].

#### Medical variables

Data on diagnosis, type of treatment, chemotherapy dose (for breast cancer only), and tumor stage were gathered from the medical records.

#### Exercise adherence

Adherence to the exercise intervention has been described previously for the Phys-Can trial [31] and was calculated separately for resistance and endurance training, and as a proportion (0–100%) of total performed exercise volume divided with total prescribed volume. I.e., resistance training was calculated as performed reps x sets x weight/prescribed reps x sets x weight and endurance training as total performed minutes at prescribed intensity/prescribed minutes at prescribed intensity.

#### Exercise stage for resistance training

Previous resistance training at baseline was assessed with the Exercise Stage Assessment Instrument (ESAI) [34], based on the Transtheoretical Model Stages of Change [35]. For the analysis, the answers were dichotomized into ‘not physically active’ and ‘physically active.’ The ‘not physically active’ category included exercise stages 1, 2, and 3 (‘pre-contemplation, ‘contemplation,’ and ‘preparation’), while the ‘physically active’ category included those in exercise stages 4 and 5 (‘action’ and ‘maintenance’).

#### Maximal oxygen uptake

Baseline maximal oxygen uptake (VO_2max_) testing was performed by walking/running to exhaustion using a modified Balke protocol [36, 37]. The test was considered valid if two out of three criteria were fulfilled: (1) the test was judged as maximal by the test personnel, (2) Borg’s Rating of Perceived Exertion [38] was ≥ 17, and (3) respiratory exchange ratio was ≥ 1.1.

### Data analysis

Due to the differences between the treatment of breast cancer and prostate cancer, these diagnosis groups were analyzed separately. The outcome was the percentage change in muscle strength from baseline to follow-up. Moderators of the effect of exercise intensity (HI and LMI) on strength changes were treatment type (i.e., chemotherapy yes/no for breast cancer, ADT yes/no for prostate cancer) and inflammation (IL6 and TNFα) at follow-up after the finished exercise intervention (Fig. 1). Models with chemotherapy/ ADT as moderators were adjusted for (control variables) previous resistance exercise, age, adherence to resistance training, and baseline inflammation (IL6 and TNFα). Models with inflammation as moderators were adjusted for previous resistance exercise, age, adherence to resistance training, and chemotherapy/ADT (yes/no). All regression models were estimated with and without interaction terms. The alpha level was set to *p* ≤ 0.05 in the analyses, which, combined with the 95% CI, was used to indicate the statistical significance in the regression models. Mplus version 8.7 [39] and the robust maximum likelihood estimator were used to estimate the regression models. Missing data were handled using multiple imputations and 100 imputed datasets (see Asparouhov & Muthén, 2010 for a detailed description [40]). Furthermore, baseline comparisons regarding age, living conditions, lifestyle variables, and treatment were analyzed with independent t-test for continuous variables, Chi-square test for categorical variables, and Mann Whitney U-test for rank/ordinal scale variables (Table 1 and Additional file 1: Table S1).

**Table 1:** Participants characteristics

	Breast cancer (n = 286)		*p* value	Prostate cancer (n = 65)		*p* value
	LMI (n = 150)	HI (n = 136)		LMI (n = 33)	HI (n = 32)	
Age, years	57 ± 11	56 ± 11	.349	70 ± 6	71 ± 5	.385
*Living situation*						
Partner, n (%)	120 (81.7)	110 (82.7)		29 (93.5)	28 (90.3)	
Without partner, n (%)	27 (6.5)	23 (17.3)		2 (6.5)	3 (9.7)	
*Education*			.387			.925
University, n (%)	100 (66.7)	84 (61.8)		15 (48.4)	15 (46.9)	
Not University, n (%)	50 (33.3)	52 (38.2)		16 (48.5)	17 (51.5)	
*Tobacco use*						
Never, n (%)	85 (61.1)	76 (62.8)	.623	12 (40.0)	17 (58.6)	
Previous or less than daily use, n (%)	44 (31.9)	43 (35.5)		17 (56.7)	12 (41.4)	
Daily, n (%)	9 (6.5)	2 (1.7)		1 (3.3)	0 (0.0)	
*Alcohol use*, drinks/week						
Never or < 1, n (%)	63 (42.6)	49 (36.8)	.545	11 (35.5)	6 (19.4)	.355
1–9, n (%)	81 (54.7)	81 (60.9)		18 (58.1)	22 (71.0)	
10- > 15, n (%)	4 (2.7)	3 (2.3)		2 (6.5)	3 (9.7)	
MVPA hrs/day	1.17 ± 0.8	1.29 ± 0.8	.327	1.31 ± .84	1.5 ± 1.08	.452
VO_2_max ml/min/kg	29.9 ± 7.0	31.25 ± 7.4	.112	29.9 ± 7.4	30.0 ± 7.0	.930
BMI	25.3 ± 4.1	25.2 ± 4.4	.849	27.0 ± 4.4	26.6 ± 3.4	.721
*Exercise stage* resistance training (ESAI)			**.008**			.810
Not physically active, n (%)	108 (84.4)	83 (70.3)		20 (76.9)	20 (74.1)	
Physically active, n (%)	20 (15.6)	35 (29.7)		6 (23.1)	7 (25.9)	
*Co-morbidities*			.541			.083
No	65 (46.8)	58 (48.7)		6 (22,2)	10 (38.5)	
One co-morbidity	38 (27.3)	35 (29.4)		8 (29.6)	10 (38.5)	
Two co-morbidities	26 (18.7)	21 (17:6)		10 (37.0)	2 (7.7)	
Three or more co-morbidities	10 (7.2)	5 (4.2)		3 (11.1)	4 (15.4)	
Gleason Score Median (IQR)	Na	Na		7.0 ± 1	7.5 ± 2	.**006**
= < 7, n (%)				25 (83.3)	16 (50.0)	
> 7, n (%)				5 (16.7)	16 (50.0)	
PSA	Na	Na		7.0 (1)	7.5 (2)	.420
*TN-classification*			.375			.294
Tis	7 (5.0)	3 (2.3)		Na	Na	
T1-T2	126 (89.4)	123 (93.9)		25 (83.3)	25 (80.6)	
T3	8 (5.7)	5 (3.8)		4 (13.3)	2 (6.5)	
T4	0 (0.0)	0 (0.0)		1 (3.3)	4 (12.9)	
N1	21 (14.8)	23 (17.3)		3 (9.1)	2 (6.3)	
*Chemotherapy* n (%)	98 (65.3)	90 (66.8)	.813	Na	Na	
Capecitabine/CEX	1	7				
F/E75C	31	27				
F/E90-100C	61	52				
Docetaxcel 100	53	41				
Docetaxcel 75–80	41	33				
Radiotherapy breast, n(%)	122 (81.4)	114 (84.5)	.435	Na	Na	
Endocrine treatment breast, n (%)	117 (78.0)	96 (71.1)	.324	Na	Na	
Radiotherapy prostate	Na	Na				
Brachy therapy only, n (%)				2 (6.1)	0 (0.0)	
External radiotherapy only, n (%)				19 (57.6)	13 (40.6)	
Brachy therapy and external radiotherapy, n (%)				12 (36.4)	19 (59.4)	
ADT n (%)	Na	Na		17 (53.1)	19 (59.4)	.614

Bold font indicates statistical significance

n vary due to missing data. Data presented as mean and standard deviation unless stated otherwise

BRCA: Breast cancer. PRCA: Prostate cancer. LMI: Low-moderate intensity group. HI: High intensity group. n: number participants. SD: Standard Deviations. MVPA: Moderate to Vigorous Physical Activity. VO_2_Max: Maximal oxygen uptake. BMI: Body Mass Index. ESAI: Exercise Stage Assessment Instrument. Na: not applicable. PSA; Prostate-Specific Antigen. TN classification according to the American Joint Committee on Cancer 8 2017, T: tumor size N: lymph node status. Tis: Tumor in situ. F/EC: Fluorouracil 500–600 mg/m^2^ and/or only Epirubicin 75–100 mg/m^2^ –Cyclophosphamide 500–600 mg/m^2^; CEX: Epirubicin-Cyclophosphamide-Capecitabine; CAPOX: Capecitabine-Oxaliplatin. ADT; Androgen deprivation therapy

## Results

The characteristics of the participants included in the analysis are presented in Table 1. Of the 476 women with breast cancer and 100 men with prostate cancer participating in the Phys-Can, 286, (60.1%) and 65 (65.0%) respectively, had complete data on the outcome variable muscle strength and were included in the analysis. Missing moderator variables for breast cancer were 0.3% for chemotherapy, 25.2% for IL6, and 24.8% for TNFα. For prostate cancer, missing data for ADT were 1.5% and 16.9% for both IL6 and TNFα. Furthermore, missing control variables ranged from 1.5 to 21.7%. For breast cancer, adherence to the resistance training was 67.6% in LMI and 62.0% in HI, and for endurance training 71.8% in LMI and 53.9% in HI. For prostate cancer, adherence to the resistance training was 70.7% in LMI and 73.8% in HI, and for endurance training 64.0% in LMI and 60.0% in HI.

For women with breast cancer, IL6 (*b* = 2.469, 95% CI [− 7.614, 12.552], *p* = 0.631) and TNFα (*b* = 0.036, 95% CI [− 6.345, 6.418], *p* = 0.991) were not statistically significant moderators of the effect of exercise intensity on changes in muscle strength (Table 2, Model 1.2). A similar result was observed for chemotherapy treatment, which was not a statistically significant moderator of the effect of exercise intensity on strength change (*b* = 4.893, 95% CI [− 2.938, 12.724], *p* = 0.221; Table 2, Model 2.2). For men with prostate cancer, IL6 (*b* = − 1.423, 95% CI [− 17.894, 15.048], *p* = 0.866) and TNFα (*b* = − 1.905, 95% CI [− 8.542, 4.732], *p* = 0.574) were not statistically significant moderators of the effect of exercise intensity on changes in muscle strength (Table 3, Model 3.2). In addition, ADT was not a statistically significant moderator of the effect of exercise intensity on change in muscle strength (*b* = − 0.180, *SE* = 5.623, 95% CI [− 11.201, 10.841], *p* = 0.974; Model 4.2).

**Table 2:** Moderating effects of inflammation and treatment for patients with breast cancer (*n* = 286)

	Model 1.1 without interaction terms					Model 1.2 with interaction terms				
	*b*	*SE*	95% CI		*p*	*b*	*SE*	95% CI		*p*
			LL	UL				LL	UL	
Model 1: Moderating effects of inflammation for patients with breast cancer										
**Main effects**										
Exercise group (HI/LMI)	**7.495**	**2.121**	**3.337**	**11.654**	**< .001**	**7.714**	**2.099**	**3.601**	**11.828**	**< .001**
IL6 at follow-up	− 1.522	1.509	− 4.479	1.435	.313	− 1.775	1.617	− 4.945	1.396	.273
TNFα at follow-up	− 1.149	1.646	− 4.375	2.078	.485	− 1.235	2.076	− 5.305	2.834	.552
*Interaction effects*										
Exercise group*IL6						2.469	5.145	− 7.614	12.552	.631
Exercise group *TNFα						0.036	3.256	− 6.345	6.418	.991
**Control variables**										
Adherence to exercise	**0.139**	**0.059**	**0.023**	**0.255**	**.019**	**0.141**	**0.062**	**0.020**	**0.262**	**.022**
Previous RT exercise	1.033	2.825	− 4.505	6.570	.715	0.930	2.805	− 4.567	6.428	.740
Age	− 0.144	0.098	− 0.336	0.048	.140	− 0.146	0.098	− 0.338	0.046	.137
Chemotherapy (Y/N)	− 3.203	2.305	− 7.720	1.314	.165	− 3.104	2.285	− 7.582	1.375	.174
*R*^2^	9.9%	10.2%								

	Model 2.1 without interaction term					Model 2.2 with interaction term				
	*b*	*SE*	95% CI		*p*	*b*	95% CI		UL	*p*
			LL	UL			*SE*	LL		
Model 2: Moderating effects of chemotherapy treatment for patients with breast cancer										
**Main effects**										
Exercise group (HI/LMI)	**7.735**	**2.085**	**3.648**	**11.823**	**< .001**	4.501	3.122	− 1.617	10.620	.149
Chemotherapy (Y/N)	− 4.003	2.327	− 8.564	0.559	.085	**− 6.305**	**2.957**	**− 12.100**	**− 0.509**	**.033**
*Interaction effects*										
Grp*Chemotherapy						4.893	3.995	− 2.938	12.724	.221
**Control variables**										
IL6 at baseline	− 0.237	1.569	− 3.366	2.892	.882	− 0.084	1.625	− 3.269	3.102	.959
TNFα at baseline	− 2.269	1.331	− 4.877	0.339	.088	− 2.270	1.327	− 4.870	0.331	.087
Adherence to exercise	**0.141**	**0.058**	**0.028**	**0.254**	**.014**	**0.136**	**0.058**	**0.023**	**0.249**	**.018**
Previous RT exercise	1.342	2.813	− 4.172	6.856	.633	1.277	2.847	− 4.303	6.857	.654
Age	− 0.145	0.089	− 0.320	0.031	.106	− 0.144	0.090	− 0.320	0.032	.110
*R*^2^	10.2%	10.6%								

Bold font indicates statistical significance

Control variables only predict the outcome. HI: high intensity. LMI: low-to-moderate intensity. CI: confidence intervals. LL: Lower limit. UL: Upper limit. IL6: Interleukin 6. TNFα: Tumor Necrosis Factor-alpha. Grp: Exercise group. RT: Resistance training. Y/N: Yes/No

**Table 3:** Moderating effects of inflammation and treatment for patients with prostate cancer (*n* = 65)

	Model 3.1 without interaction terms					Model 3.2 with interaction terms				
	*b*	*SE*	95% CI		*p*	*b*	*SE*	95% CI		*p*
			LL	UL				LL	UL	
Model 3: Moderating effects of inflammation for patients with prostate cancer										
**Main effects**										
Exercise group (HI/LMI)	1.502	2.864	− 4.111	7.114	.600	1.779	2.888	− 3.881	7.438	.538
IL6 at follow-up	1.490	4.199	− 6.740	9.720	.723	2.216	4.167	− 5.952	10.384	.595
TNFα at follow-up	**5.140**	**2.222**	**0.784**	**0.482**	**.021**	**6.796**	**2.864**	**1.182**	**12.409**	**.018**
*Interaction effects*										
Exercise group*IL6						− 1.423	8.404	− 17.894	15.048	.866
Exercise group *TNFα						− 1.905	3.386	− 8.542	4.732	.574
**Control variables**										
Adherence to exercise	**0.295**	**0.095**	**0.108**	**0.482**	**.002**	**0.307**	**0.101**	**0.110**	**0.504**	**.002**
Previous RT exercise	− 4.943	3.365	11.537	1.652	.142	− 6.150	3.228	− 12.477	0.178	.057
Age	− 0.492	0.281	− 1.043	0.058	.080	− 0.41	0.286	− 1.101	0.019	.058
ADT (Y/N)	**− 10.129**	**2.908**	**− 15.827**	**− 4.430**	**< .001**	**− 10.874**	**2.863**	**− 16.486**	**− 5.262**	**< .001**
*R*^2^	27.0%	29.8%								

	Model 4.1 without interaction term					Model 4.2 with interaction term				
	*b*	*SE*	95% CI		*p*	*b*	*SE*	95% CI		*p*
			LL	UL				LL	UL	
Model 4: Moderating effects of ADT for patients with prostate cancer										
**Main effects**										
Exercise group (HI/LMI)	1.351	2.741	− 4.021	6.722	.622	1.506	4.270	− 6.862	9.875	.724
ADT (Y/N)	**− 9.464**	**2.972**	**− 15.289**	**− 3.638**	**.001**	**− 9.198**	**3.797**	**− 16.640**	**− 1.755**	**.015**
*Interaction effects*										
Exercise group*ADT						− 0.180	5.623	− 11.201	10.841	.974
**Control variables**										
IL6 at baseline	− 0.863	1.966	− 4.717	2.990	.661	− 0.752	1.928	− 4.531	3.026	.696
TNFα at baseline	3.727	2.535	− 1.243	8.696	.142	3.760	2.413	− 0.970	8.489	.119
Adherence to exercise	**0.286**	**0.096**	**0.097**	**0.474**	**.003**	**0.284**	**0.097**	**0.093**	**0.475**	**.004**
Previous RT exercise	− 4.193	3.421	− 10.898	2.512	.220	− 3.839	3.422	− 10.546	2.867	.262
Age	− 0.317	0.281	− 0.868	0.234	.259	− 0.324	0.277	− 0.867	0.220	.243
*R*^2^	23.6%	23.6%								

Bold font indicates statistical significance

Control variables only predict the outcome. HI: high intensity. LMI: low-to-moderate intensity. CI: confidence intervals. ADT: Androgen deprivation therapy. IL6: Interleukin 6. TNFα: Tumor Necrosis Factor-alpha. Y/N: Yes/No. RT: Resistance training

For comparison of participants included in the analysis and those not included, see Additional file 1: Table S1. In short, for breast cancer patients, a larger proportion of patients with incomplete muscle strength data had more co-morbidities, they had a lower VO_2max,_ and were randomized to the high-intensity exercise group (Additional file 1: Table S1). For prostate cancer patients, patients with incomplete muscle strength data were younger and stronger (Additional file 1: Table S1).

## Discussion

In the present study, we investigated if markers of inflammation and type of neo- or adjuvant cancer treatment moderated the effects of exercise intensity on strength changes in patients with breast or prostate cancer. The primary finding of our study was that neither inflammation, chemotherapy, nor ADT had a moderating effect of exercise intensity on strength changes in patients with breast or prostate cancer.

Cytokines, such as chronic low-grade levels of TNFα and IL6, are associated with muscle atrophy and contractile dysfunction of skeletal muscle [20, 21]. In our population, we observed elevations in IL6 and TNFα during adjuvant treatment (at the end of chemotherapy and radiotherapy treatment) [32]; thus, we hypothesized that this increase would negatively impact the response to exercise on muscle strength. However, this was not observed for either of the exercise intensities. In older populations, where higher levels of inflammation are common [41], exercise has been shown to reduce inflammation [42, 43]. In previously published results from the Phys-Can trial, the observed elevations in IL6 and TNFα during adjuvant treatment in our population were reduced back to the baseline levels at our 6-month measurements among those participating in the HI exercise. Small reductions were also observed among participants in the LMI group, albeit not to the same extent as in the HI group [33]. This reduction in inflammation in response to exercise, however, may have been sufficient to protect the skeletal muscle from the negative effects that are observed from increased levels of inflammation. Furthermore, the effect of exercise on low-grade inflammation is believed to be caused by increased energy expenditure and acute increase in myokines and, therefore, may be more dependent on exercise volume than exercise intensity per se [44]. In addition, we also need to consider the possibility that the levels of inflammation (baseline and follow-up) may have been too low to affect the training response negatively [45].

Treatment with doxorubicin and paclitaxel chemotherapy has previously been demonstrated to cause atrophy of muscle fibers and mitochondrial dysfunction in women with breast cancer [11], and combined aerobic and resistance training has been found to counteract some of these negative effects [12]. Somewhat surprisingly, the results from our study suggest that chemotherapy does not moderate the effects of exercise intensity on muscle strength in our population; instead, it seems that exercise for women with breast cancer at both low-moderate and high intensity provides strength gains proportional to the intensity. This indicates that although the training effects may be muted by chemotherapy, the trainability remains intact. Similarly, ADT for prostate cancer is known to negatively affect muscle strength [41]; however, our findings suggest that this type of treatment does not have a moderating effect of exercise intensity on muscle strength. This finding is contrary to our hypothesis that the HI group, by providing a higher muscular stimulus, would be less affected by ADT treatment than the LMI group. However, because our participants received the same volume of resistance training, which is in line with recommendations for the healthy population [46], this stimulus may have been sufficient to provide similar protective effects of resistance training corresponding to the intensity (i.e., training load). Nonetheless, the optimal exercise volume for counteracting the negative effects of ADT is unknown. Furthermore, these results should be interpreted with caution since we only included 65 men with prostate cancer in the analysis.

In the present study, we also observed some direct effects. For instance, ADT therapy for men with prostate cancer seems to contribute to a decrease in strength regardless of exercise intensity (Table 3, Model 4.1). Previous research has shown that, when comparing men treated with ADT to a control group, men on ADT were about 12% weaker in the lower body [47]. Treatment with ADT reduces lean mass by about 2–4% during the first year of therapy [15], which could partly explain the lower levels of muscle strength. Although resistance training can counteract this negative effect [48], the timing of the intervention (e.g., initiating exercise after the start of ADT) may affect the trainability of the skeletal muscles negatively through ADT imposed inhibition of satellite cell response [49]. In our study, the patients with prostate cancer were recruited at the oncology department before starting radiotherapy; however, the ADT treatment, in many cases, was initiated at the urological department several weeks prior. Hence, most of the participants had already started ADT when beginning the exercise intervention.

Not surprisingly, the findings in our study clearly showed that adherence to the exercise program is a significant predictor of improvements in muscle strength, not only for women with breast cancer but also for men with prostate cancer (Tables 2–3). Therefore, focusing on helping patients to maintain a high adherence to an exercise program may be of more importance than focusing on a high training load. Moreover, our results show that participating in high-intensity exercise predicts a greater increase in strength for women with breast cancer compared to low-moderate intensity.

### Strengths and limitations

The present study is based on data from a large RCT where participants received supervised resistance training with rigorous control of the resistance training volume [30]. Furthermore, the outcome of muscle strength was assessed with 1 RM, which is considered a valid means to assess muscle strength in humans [50] moreover, the participants received a 6-week familiarization period which further enhances the test’s reliability. However, the aim of the RCT was to investigate the effects of an exercise intervention on cancer related fatigue. Therefore, power calculations were performed to facilitate the analysis of the main outcome, and not for the analysis presented herein. As such, results from this present study should be interpreted with caution, as some of the analyses included a relatively small sample (e.g., patients with prostate cancer).

We only included participants with complete data on the outcome, muscle strength. A baseline comparison was made between participants included in the analysis and those who were excluded, but only a few differences were observed between the two groups (Additional file 1: Table S1). Moreover, baseline comparisons between the exercising groups (HI vs. LMI) of the included participants revealed that a higher proportion of participants with breast cancer in the HI group were already actively participating in resistance training, however all models for both breast and prostate cancer were adjusted for previous resistance training. However, there was no difference between the groups with regard to physical activity level and VO_2max_, indicating that the two groups were similar regarding their current physical activity status. In men with prostate cancer, participants in the HI group had a higher median Gleason score, and a higher proportion of men in the HI group received combined brachy and external radiotherapy, which is associated with more side-effects. Yet, there was no statistically significant difference in the proportion of participants receiving ADT (Table 1). Our participants were relatively healthy individuals receiving neo- or adjuvant chemotherapy (breast cancer) or ADT (prostate cancer) treatment; thus, the results from this study may not be generalized to a population with metastasized cancer or receiving other treatment regimes.

## Conclusion

The findings in the present study are that TNF-alpha, IL6, and type of treatment do not moderate the effects of exercise intensity on strength. Therefore, patients can be recommended to engage in high-intensity exercise for superior effect on strength gain, regardless of having high levels of inflammation or receiving chemotherapy or ADT.

## Supplementary Information

**Additional file 1**.** Supplementary Table 1**. Baseline characteristics of participants included in the analysis vs. participants not included.

## Data Availability

The datasets generated and/or analyzed during the current study are not publicly available since the participants did not provide consent to share the data openly; however, de-identified datasets used are available from the corresponding author upon reasonable request.

## Abbreviations

ADT: Androgen deprivation therapy
BRCA: Breast cancer
CI: Confidence interval
HI: High intensity exercise
IL-6: Interleukin-6
LMI: Low-to-moderate intensity
PRCA: Prostate cancer
RCT: Randomized controlled trial
RM: Repetition maximum
TNFα: Tumor necrosis factor-alpha
VO_2max_: Maximal oxygen uptake

## References

[1] Arnold M, Rutherford MJ, Bardot A, Ferlay J, Andersson TM, Myklebust TA (2019). Progress in cancer survival, mortality, and incidence in seven high-income countries 1995–2014 (ICBP SURVMARK-2): a population-based study. Lancet Oncol.

[2] Stein KD, Syrjala KL, Andrykowski MA (2008). Physical and psychological long-term and late effects of cancer. Cancer.

[3] Bylow K, Mohile SG, Stadler WM, Dale W (2007). Does androgen-deprivation therapy accelerate the development of frailty in older men with prostate cancer?: a conceptual review. Cancer.

[4] Villasenor A, Ballard-Barbash R, Baumgartner K, Baumgartner R, Bernstein L, McTiernan A (2012). Prevalence and prognostic effect of sarcopenia in breast cancer survivors: the HEAL Study. J Cancer Surviv.

[5] Cruz-Jentoft AJ, Bahat G, Bauer J, Boirie Y, Bruyere O, Cederholm T (2019). Sarcopenia: revised European consensus on definition and diagnosis. Age Ageing.

[6] Ostir GV, Kuo YF, Berges IM, Markides KS, Ottenbacher KJ (2007). Measures of lower body function and risk of mortality over 7 years of follow-up. Am J Epidemiol.

[7] Hayward R, Hydock D, Gibson N, Greufe S, Bredahl E, Parry T (2013). Tissue retention of doxorubicin and its effects on cardiac, smooth, and skeletal muscle function. J Physiol Biochem.

[8] Hydock DS, Lien CY, Jensen BT, Schneider CM, Hayward R (2011). Characterization of the effect of in vivo doxorubicin treatment on skeletal muscle function in the rat. Anticancer Res.

[9] van Norren K, van Helvoort A, Argiles JM, van Tuijl S, Arts K, Gorselink M (2009). Direct effects of doxorubicin on skeletal muscle contribute to fatigue. Br J Cancer.

[10] Gilliam LAA, Fisher-Wellman KH, Lin CT, Maples JM, Cathey BL, Neufer PD (2013). The anticancer agent doxorubicin disrupts mitochondrial energy metabolism and redox balance in skeletal muscle. Free Radic Biol Med.

[11] Guigni BA, Callahan DM, Tourville TW, Miller MS, Fiske B, Voigt T (2018). Skeletal muscle atrophy and dysfunction in breast cancer patients: role for chemotherapy-derived oxidant stress. Am J Physiol Cell Physiol.

[12] Mijwel S, Cardinale DA, Norrbom J, Chapman M, Ivarsson N, Wengstrom Y (2018). Exercise training during chemotherapy preserves skeletal muscle fiber area, capillarization, and mitochondrial content in patients with breast cancer. FASEB J.

[13] Mottet N, Bellmunt J, Bolla M, Briers E, Cumberbatch MG, De Santis M (2017). EAU-ESTRO-SIOG guidelines on prostate cancer. Part 1: screening, diagnosis, and local treatment with curative intent. Eur Urol.

[14] Spratt DE, Malone S, Roy S, Grimes S, Eapen L, Morgan SC (2021). Prostate radiotherapy with adjuvant androgen deprivation therapy (ADT) improves metastasis-free survival compared to neoadjuvant ADT: an individual patient meta-analysis. J Clin Oncol.

[15] Smith MR, Saad F, Egerdie B, Sieber PR, Tammela TL, Ke C (2012). Sarcopenia during androgen-deprivation therapy for prostate cancer. J Clin Oncol.

[16] Langley RE, Price P, Abel PD. Re: Claude C. Schulman, Jacques Irani, Juan Morote, et al. Androgen-deprivation therapy in prostate cancer: a European expert panel review. Eur Urol suppl 2010;9:675–91. Eur Urol. 2011;59(4):e24–5; author reply e6. 10.1016/j.eururo.2011.01.005 21255905

[17] Mantovani A, Allavena P, Sica A, Balkwill F (2008). Cancer-related inflammation. Nature.

[18] Balkwill F, Mantovani A (2001). Inflammation and cancer: back to Virchow?. Lancet.

[19] Tuttle CSL, Thang LAN, Maier AB (2020). Markers of inflammation and their association with muscle strength and mass: a systematic review and meta-analysis. Ageing Res Rev.

[20] Haddad F, Zaldivar F, Cooper DM, Adams GR. IL-6-induced skeletal muscle atrophy. J Appl Physiol (1985). 2005;98(3):911–7. 10.1152/japplphysiol.01026.2004 15542570

[21] Li X, Moody MR, Engel D, Walker S, Clubb FJ, Sivasubramanian N (2000). Cardiac-specific overexpression of tumor necrosis factor-alpha causes oxidative stress and contractile dysfunction in mouse diaphragm. Circulation.

[22] Wahlin-Larsson B, Wilkinson DJ, Strandberg E, Hosford-Donovan A, Atherton PJ, Kadi F (2017). Mechanistic links underlying the impact of C-reactive protein on muscle mass in elderly. Cell Physiol Biochem.

[23] Puetz TW, Herring MP (2012). Differential effects of exercise on cancer-related fatigue during and following treatment: a meta-analysis. Am J Prev Med.

[24] Adams SC, Segal RJ, McKenzie DC, Vallerand JR, Morielli AR, Mackey JR (2016). Impact of resistance and aerobic exercise on sarcopenia and dynapenia in breast cancer patients receiving adjuvant chemotherapy: a multicenter randomized controlled trial. Breast Cancer Res Treat.

[25] Hiensch AE, Mijwel S, Bargiela D, Wengstrom Y, May AM, Rundqvist H. Inflammation Mediates Exercise Effects on Fatigue in Patients with Breast Cancer. Med Sci Sports Exerc. 2020. 10.1249/MSS.0000000000002490 PMC7886356 32910094

[26] Koeppel M, Mathis K, Schmitz KH, Wiskemann J (2021). Muscle hypertrophy in cancer patients and survivors via strength training. A meta-analysis and meta-regression. Crit Rev Oncol Hematol.

[27] Sweegers MG, Altenburg TM, Brug J, May AM, van Vulpen JK, Aaronson NK (2019). Effects and moderators of exercise on muscle strength, muscle function and aerobic fitness in patients with cancer: a meta-analysis of individual patient data. Br J Sports Med.

[28] Lopez P, Radaelli R, Taaffe DR, Newton RU, Galvao DA, Trajano GS (2021). Resistance training load effects on muscle hypertrophy and strength gain: systematic review and network meta-analysis. Med Sci Sports Exerc.

[29] Grgic J, Schoenfeld BJ, Davies TB, Lazinica B, Krieger JW, Pedisic Z (2018). Effect of resistance training frequency on gains in muscular strength: a systematic review and meta-analysis. Sports Med.

[30] Demmelmaier I, Brooke HL, Henriksson A, Mazzoni AS, Bjorke ACH, Igelstrom H (2021). Does exercise intensity matter for fatigue during (neo-)adjuvant cancer treatment? The Phys-Can randomized clinical trial. Scand J Med Sci Sports.

[31] Mazzoni AS, Brooke HL, Berntsen S, Nordin K, Demmelmaier I (2020). Exercise adherence and effect of self-regulatory behavior change techniques in patients undergoing curative cancer treatment: secondary analysis from the phys-can randomized controlled trial. Integr Cancer Ther.

[32] Schumann M, Feuerbacher JF, Sunkeler M, Freitag N, Ronnestad BR, Doma K (2022). Compatibility of concurrent aerobic and strength training for skeletal muscle size and function: an updated systematic review and meta-analysis. Sports Med.

[33] Schauer T, Mazzoni AS, Henriksson A, Demmelmaier I, Berntsen S, Raastad T, et al. Exercise intensity and markers of inflammation during and after (neo-) adjuvant cancer treatment. Endocr Relat Cancer. 2021. 10.1530/ERC-20-0507 33608485

[34] Nigg C, Riebe D. The transtheoretical model: research review of exercise behavior in older adults. In: Burbank P, Riebe D, editors. Promoting exercise and behavior change in older adults: interventions with the transtheoretical model New York: Springer; 2002. p. 147–80.

[35] Prochaska JO, DiClemente C, Norcross JC (1992). In search of how people change. Am Psychol.

[36] Edvardsen E, Hansen BH, Holme IM, Dyrstad SM, Anderssen SA (2013). Reference values for cardiorespiratory response and fitness on the treadmill in a 20- to 85-year-old population. Chest.

[37] Berntsen S, Aaronson NK, Buffart L, Borjeson S, Demmelmaier I, Hellbom M (2017). Design of a randomized controlled trial of physical training and cancer (Phys-Can) - the impact of exercise intensity on cancer related fatigue, quality of life and disease outcome. BMC Cancer.

[38] Borg GAV (1970). Perceived exertion as an indicator of somatic stress. Scand J Rehabil Med.

[39] Muthén Ma. Mplus User’s Guide. Los Angeles1998–2017.

[40] Asparouhov T, Muthén, B. Multiple imputation with Mplus: Technical implementation http://statmodel2.com/download/Imputations7.pdf 2010

[41] Schaap LA, Pluijm SM, Deeg DJ, Harris TB, Kritchevsky SB, Newman AB (2009). Higher inflammatory marker levels in older persons: associations with 5-year change in muscle mass and muscle strength. J Gerontol A Biol Sci Med Sci.

[42] Woods JA, Wilund KR, Martin SA, Kistler BM (2012). Exercise, inflammation and aging. Aging Dis.

[43] Zheng G, Qiu P, Xia R, Lin H, Ye B, Tao J (2019). Effect of aerobic exercise on inflammatory markers in healthy middle-aged and older adults: a systematic review and meta-analysis of randomized controlled trials. Front Aging Neurosci.

[44] Pedersen BK (2011). Muscles and their myokines. J Exp Biol.

[45] Rose GL, Skinner TL, Mielke GI, Schaumberg MA (2021). The effect of exercise intensity on chronic inflammation: a systematic review and meta-analysis. J Sci Med Sport.

[46] Bull FC, Al-Ansari SS, Biddle S, Borodulin K, Buman MP, Cardon G (2020). World Health Organization 2020 guidelines on physical activity and sedentary behaviour. Br J Sports Med.

[47] Galvao DA, Taaffe DR, Spry N, Joseph D, Turner D, Newton RU (2009). Reduced muscle strength and functional performance in men with prostate cancer undergoing androgen suppression: a comprehensive cross-sectional investigation. Prostate Cancer Prostatic Dis.

[48] Nilsen TS, Johansen SH, Thorsen L, Fairman CM, Wisloff T, Raastad T (2022). Does androgen deprivation for prostate cancer affect normal adaptation to resistance exercise?. Int J Environ Res Public Health.

[49] Chen Z, Zhang Y, Lu C, Zeng H, Schumann M, Cheng S (2019). Supervised physical training enhances muscle strength but not muscle mass in prostate cancer patients undergoing androgen deprivation therapy: a systematic review and meta-analysis. Front Physiol.

[50] Verdijk LB, van Loon L, Meijer K, Savelberg HH (2009). One-repetition maximum strength test represents a valid means to assess leg strength in vivo in humans. J Sports Sci.

