# Supplementary material for: Does inflammation markers or treatment type moderate exercise intensity effects on changes in muscle strength in cancer survivors participating in a 6-month combined resistance- and endurance exercise program? Results from the Phys-Can trial
Source: BMC Sports Sci Med Rehabil. 2023 Jan 19;15:8. doi: 10.1186/s13102-023-00617-3 (PMC9854232; doi:10.1186/s13102-023-00617-3)
Supplement: Supplementary file 1 — Additional file 1. Supplementary Table 1. Baseline characteristics of participants included in the analysis vs. participants not included. [file 13102_2023_617_MOESM1_ESM.docx]

**Supplementary Table 1. Baseline characteristics of participants included in the analysis vs. participants not included**

|  | Breast cancer | |  | Prostate cancer | |  |
| --- | --- | --- | --- | --- | --- | --- |
|  | Included | Not included |  | Included | Not included |  |
|  | (n=286) | (n=190) | *P-value* | (n=65) | (n=35) | *P-value* |
|  |  |  |  |  |  |  |
| Age, years | 55.9 ± 11.3 | 56.8 ± 12.2 | .444 | 70.4 ± 5.4 | 67.5 ± 7.5 | **.023** |
| Living situation |  |  |  |  |  |  |
| Partner, n (%) | 230 (82) | 130 (77) | .178 | 57 (92) | 24 (80) | .208 |
| Without partner, n (%) | 50 (18) | 38 (22) |  | 5 (8) | 6 (20) |  |
| Education level |  |  | .072 |  |  | .830 |
| University, n (%) | 184 (66) | 103 (61) |  | 30 (38) | 15 (50) |  |
| Not University, n (%) | 96 (34) | 67 (39) |  | 48 (62) | 15 (50) |  |
| Chemotherapy |  |  | .310 |  |  |  |
| No, n (%) | 97 (34) | 57 (39) |  |  |  |  |
| Yes, n (%) | 188 (66) | 90 (61) |  |  |  |  |
| ADT |  |  |  |  |  | .812 |
| No, n (%) |  |  |  | 28 (44) | 13 (46) |  |
| Yes, n (%) |  |  |  | 36 (56) | 15 (54) |  |
| Comorbidities |  |  | **.024** |  |  | .788 |
| No, n (%) | 123 (48) | 56 (36) |  | 16 (30) | 7 (32) |  |
| Yes (1–3+), n (%) | 135 (52) | 99 (64) |  | 37 (70) | 15 (68) |  |
| Prescribed exercise intensity |  |  | **.001** |  |  | .053 |
| High, n (%) | 136 (48) | 92 (54) |  | 32 (49) | 17 (53) |  |
| Low-to-moderate, n (%) | 150 (52) | 79 (46) |  | 33 (51) | 15 (47) |  |
| Physical fitness |  |  |  |  |  |  |
| VO2max, ml/kg/min^-1^ | 30.5 ± 7.2 | 28.4 ± 7.2 | **.003** | 29.9 ± 7.1 | 29.7 ± 8.4 | .904 |
| Tot. muscle strength, Kg | 134.3 ± 41.2 | 133.3 ± 41.7 | .856 | 196.8 ± 43.4 | 221.4 ± 54.4 | **.034** |
| Exercise stage, (ESAI)  Strength training |  |  | .326 |  |  | *.*743 |
| Not physically active  (Stage 1–3), n (%) | 191 (78) | 113 (82) |  | 58 (74) | 18 (72) |  |
| Physically active  (Stage 4–5), n (%) | 55 (22) | 25 (18) |  | 20 (26) | 7 (28) |  |
| Only participants with complete strength test data were included in the analysis. n vary due to missing data. Data presented as mean and standard deviation unless stated otherwise. ESAI: Exercise Stage Assessment Instrument. Bold font indicates statistical significance. | | | | | | |
